# Assessing the efficacy of fathead minnows (*Pimephales promelas*) for mosquito control

**DOI:** 10.1371/journal.pone.0194304

**Published:** 2018-04-12

**Authors:** Ryan T. Watchorn, Thomas Maechtle, Bradley C. Fedy

**Affiliations:** 1 School of Environment, Resources and Sustainability, University of Waterloo, Waterloo, Ontario, Canada; 2 Big Horn Environmental Consultants, Sheridan, Wyoming, United States of America; Aberystwyth University, UNITED KINGDOM

## Abstract

Mosquitoes function as important vectors for many diseases globally and can have substantial negative economic, environmental, and health impacts. Specifically, West Nile virus (WNv) is a significant and increasing threat to wildlife populations and human health throughout North America. Mosquito control is an important means of controlling the spread of WNv, as the virus is primarily spread between avian and mosquito vectors. This is of particular concern for avian host species such as the Greater sage-grouse (*Centrocercus urophasianus*), in which WNv negatively impacts fitness parameters. Most mosquito control methods focus on the larval stages. In North America, control efforts are largely limited to larvicides, which require repeated application and have potentially negative ecological impacts. There are multiple potential advantages to using indigenous fish species as an alternative for larval control including lowered environmental impact, decreased costs in terms of time and financial inputs, and the potential for the establishment of self-sustaining fish populations. We tested the efficacy of using fathead minnows (*Pimephales promelas*) as biological control for mosquito populations in livestock reservoirs of semiarid rangelands. We introduced minnows into 10 treatment reservoirs and monitored an additional 6 non-treated reservoirs as controls over 3 years. Adult mosquitoes of species known to transmit WNv (e.g., *Culex tarsalis*) were captured at each site and mosquito larvae were also present at all sites. Stable isotope analysis confirmed that introduced fathead minnows were feeding at the mosquito larvae trophic level in all but one treatment pond. Treatment ponds demonstrated suppressed levels of mosquito larva over each season compared to controls with a model-predicted 114% decrease in larva density within treatment ponds. Minnows established self-sustaining populations throughout the study in all reservoirs that maintained sufficient water levels. Minnow survival was not influenced by water quality. Though minnows did not completely eradicate mosquito larvae, minnows are a promising alternative to controlling mosquito larvae density within reservoirs. We caution that careful site selection is critical to avoid potential negative impacts, but suggest the introduction of fathead minnows in reservoirs can dramatically reduce mosquito larva abundance and potentially help mitigate vector-borne disease transmission.

## Introduction

Mosquitoes are global pest species and the primary vectors for a variety of diseases such as Malaria, Dengue, Zika, Chikungunya, and West Nile virus (WNv) which can result in major ecological and economic consequences in disease-endemic locations [1]. WNv has become a threat in North America since its original detection in August 1999 [2]. An unexpected resurgence in neurological disease cases and infection rates associated with WNv in 2012 suggests that periodic outbreaks may be expected but difficult to predict [3]. Mosquitoes are important vectors for WNv transmission and 62 species in North America, mostly from the genus *Culex*, have the capacity to carry and transmit WNv [4].

Currently, there are 317 species of birds and over 30 non-avian hosts of the virus [2]. Host response to the virus varies from benign to severe. Greater sage-grouse (*Centrocercus urophasianus*; hereafter, sage-grouse) is a species of conservation concern that suffers severe impacts from WNv [5,6]. The virus is recognized as an important source of mortality in low and mid-elevation (< 1,500 m a.s.l.) sage-grouse populations throughout the west, resulting in potential impacts to 40% of their current range [7]. In particular, severe impacts have been documented in northeastern Wyoming [8,9]. Additionally, population viability analysis including WNv outbreaks suggested that local populations may be vulnerable to extirpation from even a single stressor, such as WNv [10]. Given the history of WNv in sage-grouse in northeastern Wyoming [8,9] and the susceptibility of sage-grouse populations in the area, this region is an ideal test case for WNv control efforts.

Effective approaches to controlling WNv must involve mosquito control. Eliminating mosquito breeding habitat, or controlling mosquito larval populations in anthropogenic water sources is crucial for reducing impacts [7,11]. Livestock reservoirs are one of the primary anthropogenic water sources that serves as breeding habitat for mosquitoes in northeastern Wyoming. Reservoirs are located in natural drainage basins that receive rain water and snow melt as well as natural spring run-off to replenish the water supply. Although some of the reservoirs are naturally occurring, most are formed by damming and burming the low side of the drainage (R. Fieldgrove, personal communication, January 27, 2016). These reservoirs create local mesic areas, which provide high quality brood rearing habitat for sage-grouse [12,13]. Mosquito control through the application of larvicides is one option. However, treating large areas with larvicides is expensive and can potentially have detrimental effects [14]. Non-target impacts to beneficial aquatic arthropods and vertebrates are the most common concerns [15]; however, impacts to the livestock that use the reservoirs should also be considered. Thus, a complementary and more cost-effective option is required in high-density sage-grouse areas where infection risk is greatest.

Various species of larvivorous fishes have been used around the world for biological control of disease vectors through trophic interactions [16–21]. Fathead minnows (*Pimephales promelas*) can function as effective biological control agents of mosquitoes in the larval stage [20] and are native to the local watershed and many others throughout the range of sage-grouse. The reproductive biology of fathead minnows is well studied; individuals mature rapidly, breed quickly, and can tolerate a wide range of environmental conditions, including wide variation in water quality conditions [22]. Additionally, fathead minnows can survive low oxygen levels—particularly of concern during winter months [23]. These life history traits likely facilitate the establishment of self-sustaining populations in a wide-range of environmental conditions. Here, we test the efficacy of using fathead minnows for mosquito control in northeastern Wyoming, in the hopes of minimizing the threat of WNv.

Our research addressed several objectives using a case-control design in which minnows were introduced to treatment livestock reservoirs in northeastern Wyoming. We monitored mosquito larva at both treatment and control reservoirs and minnow abundance within treatment sites. Our study took place over three years with widely varying moisture regimes. To assess overall efficacy we addressed multiple questions. We first determined whether adult *Culex tarsalis* were present in our study region, as this species has been indicated to be the primary vector for WNv in Wyoming [11]. Second, we used isotope analysis to address whether introduced minnows would likely feed on mosquito larvae present within reservoirs by analyzing trophic interactions. Third, we asked if minnow presence in a reservoir decreased larval abundance compared to control reservoirs? Finally, we determined if minnows could establish self-sustaining populations and whether minnow abundance, survival or efficacy were influenced by environmental (e.g., water quality, pond morphology) factors.

## Materials and methods

### Study area

**Fig 1:**
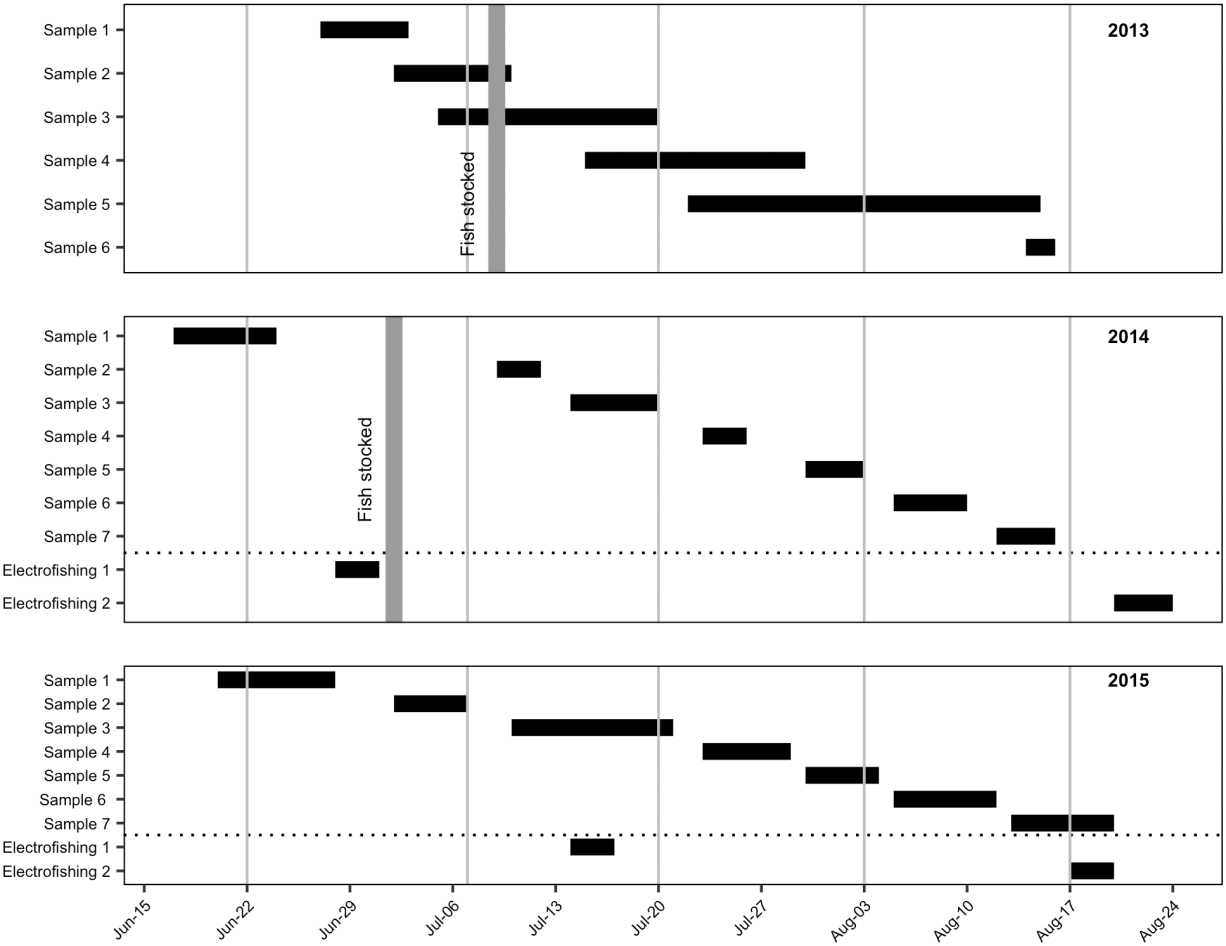
Experimental timeline. Timelines based on calendar date for each year of field season activities. Reservoirs were only sampled for mosquito larvae once during each sample period. Black horizontal lines indicate the dates during which each activity (either larvae sampling or electrofishing for minnows) was conducted. Vertical grey lines indicate when fathead minnows were stocked in treatment reservoirs in 2013 and 2015. Minnows were not introduced in 2015, but were still present in all treatment reservoirs. The dotted horizontal line separates the larva sampling and electrofishing activities in 2014 and 2015. Electrofishing did not take place in 2013. Samples for isotopic analysis were collected in 2014 during the Sample 7 period.

This study was conducted at multiple sites within the Powder River Basin in northeastern Wyoming USA (geographic location 44.7967° N, 106.9589° W). We began monitoring 15 reservoirs in 2013 and added one site in 2014. We monitored reservoirs weekly throughout the summers of 2013–2015 (Fig 1). All sites were located in arid rural environments dominated by sagebrush (*Artemesia* spp.). All reservoirs were actively used by livestock and were supplied with only naturally available water. Sites ranged in size from 0.02 to 1.33 surface hectares (ha) with a mean of 0.5 ha. Reservoir depths (at the deepest point) ranged from 0.60 to 5.2 m, with a mean of 1.72m (S1 Table). Sites were selected based on representativeness of reservoirs in the region, land access, and to ensure similar pond morphology across both treatments and controls. Sites were randomly assigned to treatment and control groups prior to larvae sampling in 2013. Simple post hoc models indicated no significant differences in size, vegetation, or depth between the treatment and control sites. All treatment reservoirs used in this study were isolated, and anthropogenic, with no other fish species present.

In 2013, adult mosquito populations were sampled bi-weekly using CO_2_ night traps to determine species diversity and abundance[24]. The night traps used a dry ice container which gradually released CO_2_ as an attractant to adult mosquitoes and were placed near the water’s edge. Individuals drawn to the trap were blown by an electric fan into a sampling net. Mosquitoes were then frozen and shipped to an entomology lab at Montana State University for identification.

Captive-raised fathead minnows were released into the treatment reservoirs (*n* = 10). The stocking rate for each site was approximately 2,500 minnows per 0.4 surface hectares (1 surface acre). We stocked 25,200 fry in 2013 and 32,500 in 2014 (Fig 1). Variation in total number of fry was due to the annual variation in pond size. Fry were not introduced to treatment reservoirs in 2015 to help isolate the effect of minnow survival and abundance in the absence of augmentation.

We quantified reservoir morphology by measuring surface area, perimeter, and maximum depth mid-season each year. We estimated near shore emergent vegetation cover as percent coverage within a circular quadrat 1 meter in diameter based on visual estimation conducted by a single observer. Vegetation cover was sampled every 10 meters along the shore and we then calculated an average percent cover for each reservoir.

### Water chemistry

Water chemistry was assessed for each treatment site. Water samples were collected from below the water surface at the deepest point of the reservoirs during Sample 3 period in 2013 and 2014 (Fig 1). Samples were delivered to the lab for analysis with in 24 hours of collections. All lab testing was conducted according to approved standard methods (SM) through the United States Environmental Protection Agency (EPA) (2015). pH was measured using the SM 4500 H B method. Alkalinity was calculated as total CaCo3, measured in mg·L^-1^ and analyzed using the SM 2320B method. Water hardness was a measure of calcium/magnesium as CaCo3 and measured in mg·L^-1^ using the SM 2340B method. Nitrogen, or ammonia, was taken as a measure of N in mg·L^-1^ using the EPA 350.1 method.

Anions and cations were measured in mg·L^-1^, using the EPA 353.2 method and EPA 200.7 method respectively. Anions were taken as a measure of Nitrate-Nitrite (as N) and cations included calcium, magnesium and sodium. Dissolved and total metals were measured in mg·L^-1^ and also followed the EPA 200.7 method. This included Iron, Magnesium, and Zinc with total metals measured by Phosphorus. Water temperature and dissolved oxygen (DO) was also recorded with a YSI meter, along with water conductivity using a handheld Hanna *combo* pH and conductivity meter. Dissolved oxygen content was monitored over the winter of 2013–2014 at accessible sites. For winter dissolved oxygen readings, a hole was drilled into the ice at the center of the reservoir and measurements were taken at 0.3m intervals beginning at the surface.

### Stable isotope analysis

Samples for isotopic analysis were collected in 2014 during the Sample 7 period (Fig 1). Mosquito larvae tissue samples were collected with dip cups. Minnows were collected using baited steel minnow traps at 5 treatment reservoirs. Five minnows were randomly selected from each reservoir and euthanized according to ethics protocols, and tissue samples were taken from side fillets of muscle and bone (not including internal organs) and placed into labeled vials with demineralised water for transport. All animal tissues were subsequently dried. Adult mosquito and larva tissue samples were placed in a drying oven for a minimum of 12 hours at 50–75 degrees Celsius. Minnow tissue samples were placed in a drying oven for a minimum of 24 hours at 50–75 degrees Celsius. Dried samples were then pulverized to a homogenate with a ceramic mortar and pestle. Approximately 0.3mg (300mcg) of prepared material was used for stable isotope analysis (SIA) completed with a Delta Plus Continuous Flow Stable Isotope Ratio Mass Spectrometer (Thermo Finnigan, Breman, Germany) coupled to a Carlo Erba elemental analyzer (CHNS-O EA1108, Carlo Erba, Millan, Italy). Analyses were conducted at the Environmental Isotope Laboratory, University of Waterloo (Waterloo, Ontario) with an analytical precision of ±0.2‰ (δ^13^C) and ±0.3‰ (δ^15^N). Samples weights necessary for SIA were obtained from a high precision ultra microbalance (Model XP2U, Mettler-Toledo GmbH, Greifensee, Switzerland).

### Population monitoring

Larva sampling was conducted using a stratified random sampling design along the reservoir shores. Samples were collected from treatment and control sites on an approximately weekly basis throughout the field seasons (Fig 1). Count data of larva and larva exoskeletons were obtained using the standard 350ml dip cup method [20,25]. Sampling was stratified based on vegetation cover. This stratification recognized that sampling efforts should be concentrated in areas where habitat characteristics such as vegetation cover would likely lead to higher densities of mosquito larva. Dip samples were taken from the water edge utilizing a *stalking-method* approach to avoid disturbance. The *stalking-method* for larva sampling required researchers to approach the water edge with as little disturbance as possible and to avoid casting shadows onto the water where samples are taken. Dip samples were taken every 10m for non-vegetated edge, every 5m for vegetated edge with emergent vegetation, and every 7.5m along vegetated edge with submersed vegetation. Where drainages lead to long shallow vegetated tails to the reservoir, sampling would be conducted 10m in to that drainage.

Presence or absence of fathead minnows was recorded weekly based on sightings and minnow trapping success using baited steel traps when sightings were inconclusive. Reproductive efforts were assessed by the presence or absence of eggs on artificial breeding substrates introduced at the beginning of the 2013 field season. Standard wooden shipping pallets were used as artificial substrates. Observations of male nesting activity were also used as indicators of reproductive effort. Relative densities were determined using standard electrofishing techniques using the Smith-Root LR-24 or HallTeck HT-2000 backpack shocker. Electrofishing protocol consisted of shocking 10% of the total reservoir perimeter between 0.5-2m from shore in the littoral zone. Total number of transects used at each site was dependent on reservoir size with each transect spanning approximately 6m (*n* transects = (Reservoir perimeter (m) * 0.10) / 6m). Each transect was shocked for 90 seconds. Personnel operating the electrofisher would begin shocking at the start of each transect, moving forward at a steady pace throughout the duration of the shocking period. Due to difficulty moving through the substrate of the reservoir, fish counts were based on observation rather than collection. Only fish *turned* by the electrofisher would be counted (*turning* a fish consisted of any fish visibly affected by the current to the point of flipping over or surfacing, or becoming temporarily stunned). Voltage and general output settings such as duty-cycle were specific to each site based on the LR-24 automatic pre-set function guided primarily by conductivity. One treatment site, T7 could not be sampled due to steep banks and littoral zone, and abundant vegetation. This site was excluded from all analysis of fish abundance.

### Data analysis

We considered any dip-cup sample that contained either live mosquito larva or exoskeletons positive for the presence of mosquitoes. We then calculated the proportion of positive dips at each site and for each sampling occasion as *X*_*positive dips*_ */ N*_*dips*_. To estimate the influence of minnows on mosquito larvae density, we developed generalized linear mixed models using the *nlme* package in R (version 3.2.2) with the proportion of positive dips at each site as the response variable. Site (pond_id) was included as a random effect (intercept) in each model to account for random variation among sites. We specified an autoregression covariance structure (AR1) to account for temporal correlations among samples within each year and included year as a fixed effect in each model. We calculated Pearson’s correlation coefficient among all continuous covariates (fixed effects, Table 1) and did not include variables correlated r > | 0.65 | in the same model. The total model set included 12 candidate models to assess the relative influence of covariates on the proportion of positive dips. Models were compared using Akaike’s information criteria adjusted for small sample sizes (AIC_c_). Model fit was assessed through inspection of model residuals. We applied the control site trend models to the initial values (*i*.*e*., sampling occasion 1) in the treatment ponds to predict the proportion of positive dips over time in the absence of a treatment. Essentially, these models predict the likely level of larva densities in the treatment ponds in the hypothetical absence of treatment, assuming these ponds would have followed the same seasonal trajectory as the control sites.

**Table 1:** Predictor variables.

Variable	Abbreviation	Description	Range
Fathead minnows	Treatment	Reservoir stocked with fathead minnows at a rate of 2,500 individuals per 0.4 ha	0 or 1
Vegetation	Veg_cover	Average percent-coverage around reservoir perimeter either by surface or emergent vegetation	0–100%
Perimeter distance	Perim	Reservoir perimeter	72.2–874.8 m
Surface hectares	SH	A measure of open-water surface hectares of a reservoir, not including marshy drainages or shallow pools	0.02–1.33 ha
Max depth	Depth	Reservoir depth measured at deepest location	0.61–5.15 m
Sample occasion	Sample	Corresponds to 1-week sampling Intervals (1–7) between July and August	1–7
Site	pond_id	Unique individual study site identifiers	

Environmental and general predictor variables and ranges quantified for each reservoir between 2013 and 2015.

We analyzed the effect of pond morphology and water chemistry on the abundance of minnows (measured as catch per unit effort) in each treatment pond using a similar modeling approach. We modeled the average number of fish per transect (rounded to the nearest integer) using Poisson linear mixed effects models including a random intercept for each pond. All covariates were standardized prior to analysis and we assessed the level of correlation among all potential covariates for both the pond morphology and water chemistry model sets. We did not include any covariates correlated r > | 0.65 | in the same model. We used AIC_c_ to rank models. Models were estimated using the *lme4* package (Bates et al. 2014, 2015) in R software.

Minnows were obtained from a state-authorized Commercial Hatchery (Pleasant Valley Fish Farm, Nebraska). Research was conducted under a Chapter 33 Permit for scientific research issued by the Wyoming Game and Fish Department (#916). Minnows were euthanized for isotope analysis by decapitation. This research was conducted in compliance with the Animals for Research Act of Ontario (Revised Statutes of Ontario), the Guide to the Care and Use of Experimental Animals from the Canadian Council on Animal Care and the University of Waterloo’s Guidelines for the Care and Use of Animals in Research and Teaching (AUPP # 13–12).

## Results

Water surface area at the reservoirs declined throughout the summer field seasons due to decreased snow melt and precipitation throughout the summer (S1 Table). Two treatment reservoirs included in the study became seasonally ephemeral and contained no open water by the end of at least one field season. Emergent vegetation near reservoir shores ranged from 0% to 100% with a mean of 25.13%. Vegetation coverage changed throughout the year and between years likely in response to inter-annual changes in water depth (S1 Table).

**Fig 2:**
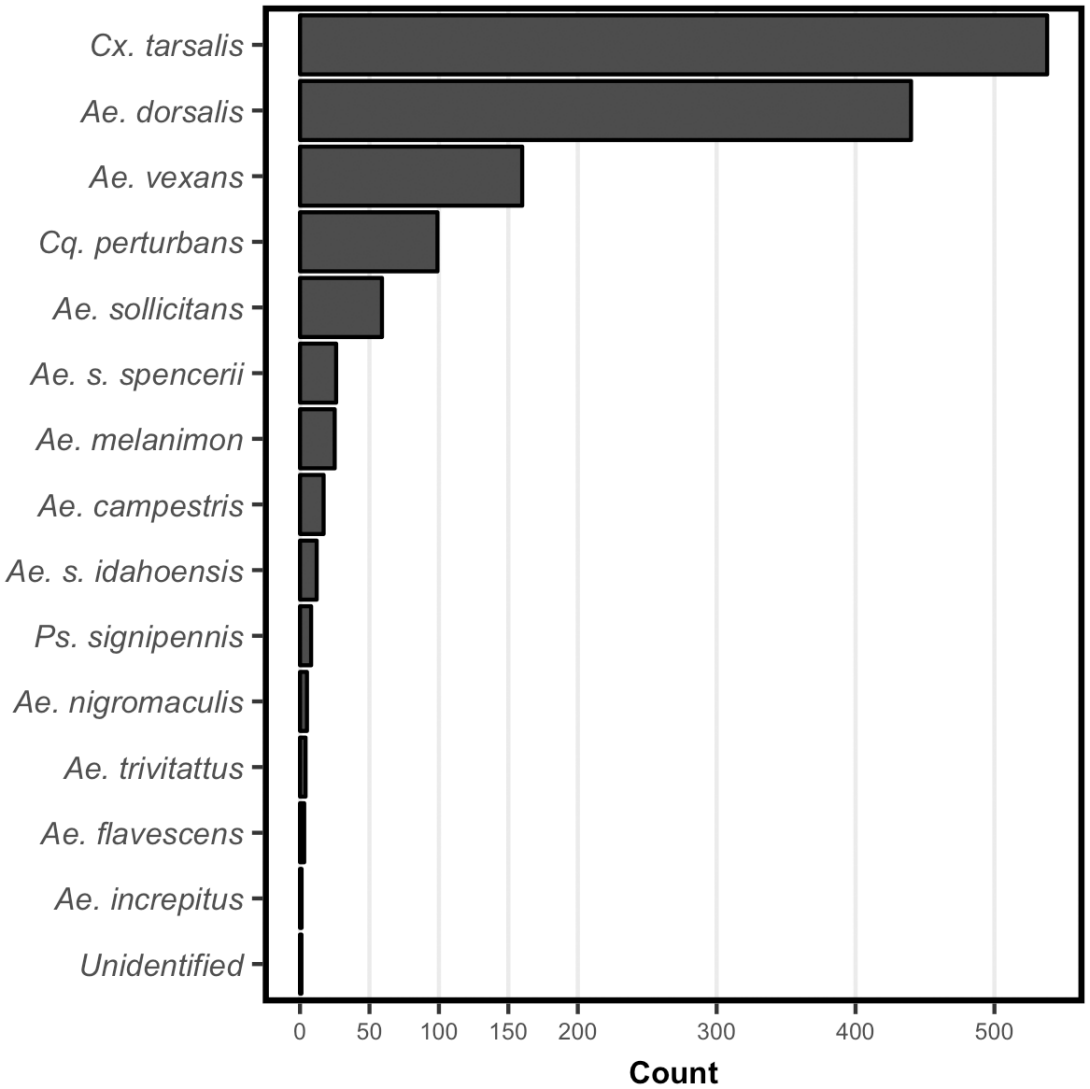
Adult moquito species. Distribution of the 1,399 individual adult mosquitoes captured using CO_2_ traps across all study reservoirs in 2013. *Culex tarsalis*, an important vector for the transmission of West Nile virus, accounted for 38% of all individuals.

We identified the mosquito species present near all reservoirs by sampling adult mosquitoes over 44 trap nights across all sites in 2013 and we captured 1,399 individuals. All but one individual could be identified to the species level. *Culex tarsalis* was the most abundance of the 14 species identified accounting for 38% of the sample population (Fig 2).

**Fig 3:**
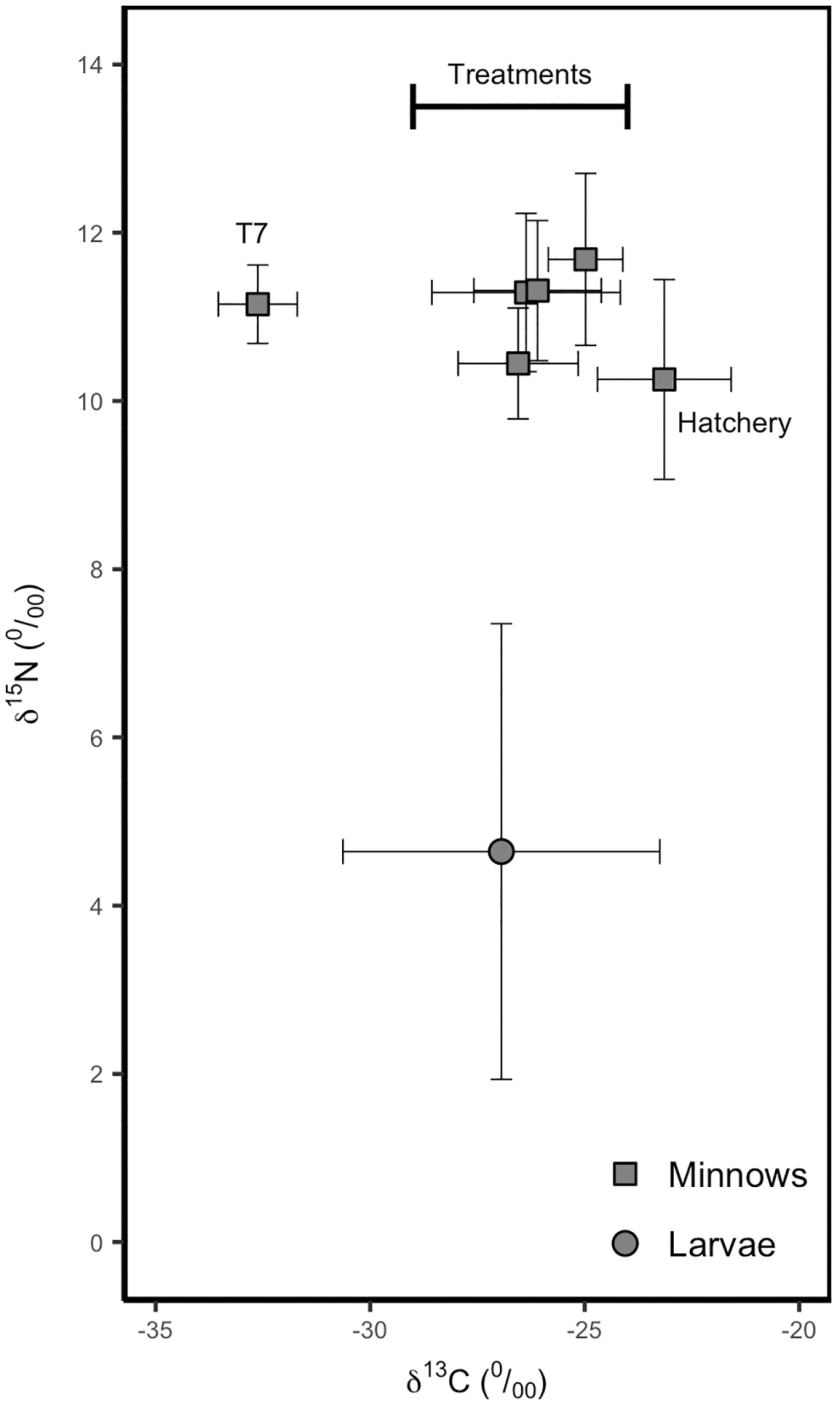
Minnow and mosquito larva isotopes. Biplot representing δ^13^C and δ^15^N stable isotope values (mean **±** SD) for minnows and mosquitos at each treatment reservoir. The Hatchery group is from minnows sampled directly from the hatchery that supplied the introduced minnows. T7 indicates one of the treatment ponds that was an outlier in both pond morphology and in the efficacy of minnows for mosquito larvae control.

Stable isotope analysis indicated predation at the predicted trophic level in all but one treatment pond [26]. Groups were summarized by site to account for variation among sites in reservoir stucture and environmental conditions, which can influence prey selection [22]. Treatment site T7, displayed a slightly lower δ ^13^C average, possibly indicating different prey selection at this site (Fig 3) [27].

**Fig 4:**
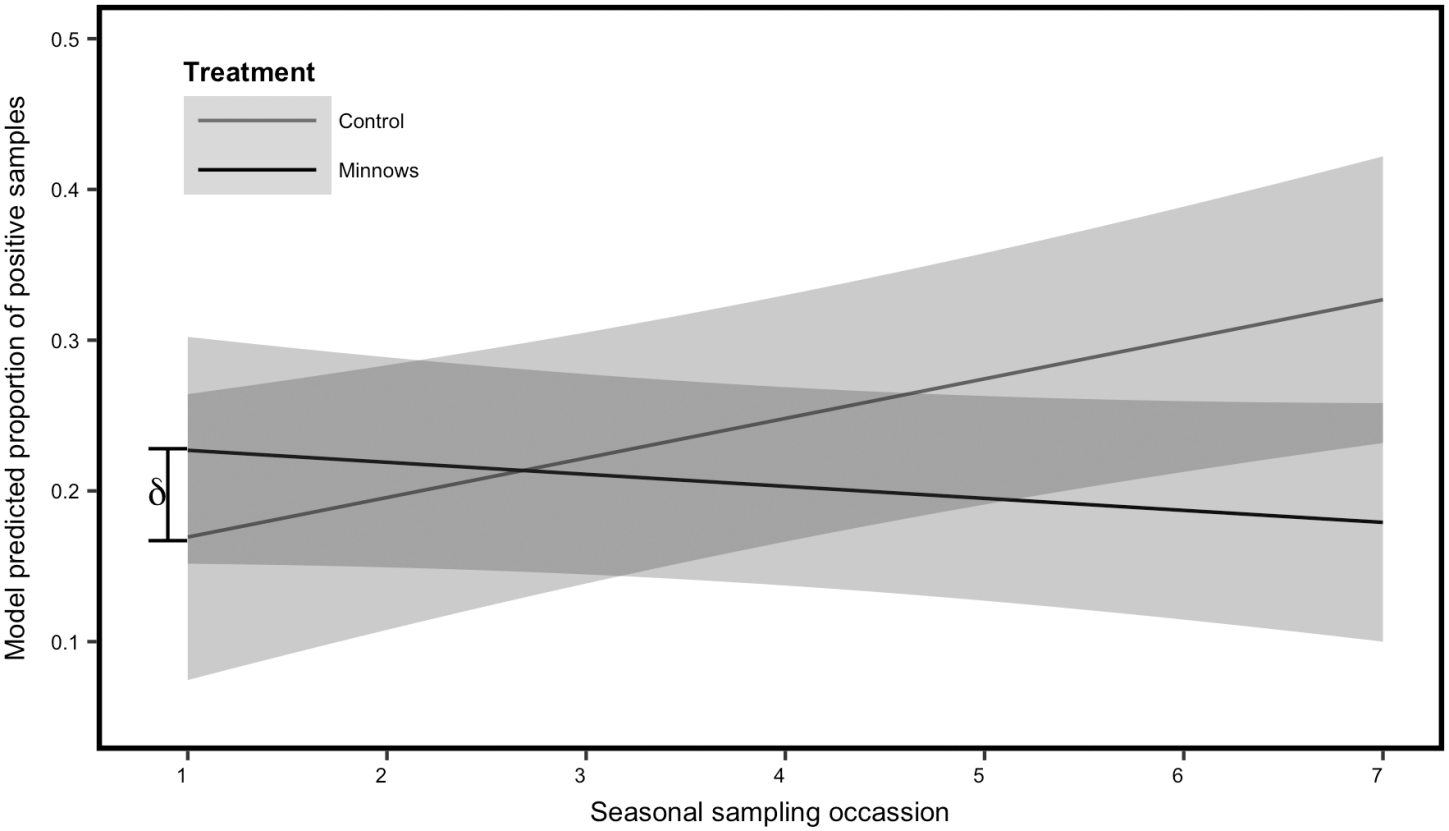
Model predicted values. Predicted values from the top linear mixed effects model predicting the proportion of samples (i.e., dips) that contained either a live mosquito larva or an exoskeleton. Data were collected from 2013–2015. Values for the control ponds (i.e., no minnows) are represented by the grey line. The black line represents the model predicted values for the treatment ponds (i.e., minnows introduced). Top model structure included a random intercept for each reservoir, a fixed covariate for year and a treatment **×** sampling occasion interaction. The model included data from 2013–2015. The difference in starting abundance between treatment and control ponds is indicated by ***δ***. The grey shading represents the 95% confidence intervals.

A total of 10,492 dip samples were collected from 2013–2015. When larvae were present in a dip sample, the number was highly skewed towards a single individual with a mode of 1 and a median of 1.75. In the initial year of the study, treatment sites appeared to display increasing larva densities up until the time of treatment. Following treatment, trajectories in larva populations declined in treatment sites while a continued seasonal increase was documented at control sites. Treatment sites were assigned at random and therefore the initial lower levels of larva at control sites was due to chance differences between the control and treatment sites (Fig 4). Combined data from the 2013–2015 seasons revealed patterns of increasing mosquito larvae densities in the control ponds. These patterns were suppressed in response to minnow treatment (Fig 4).

**Fig 5:**
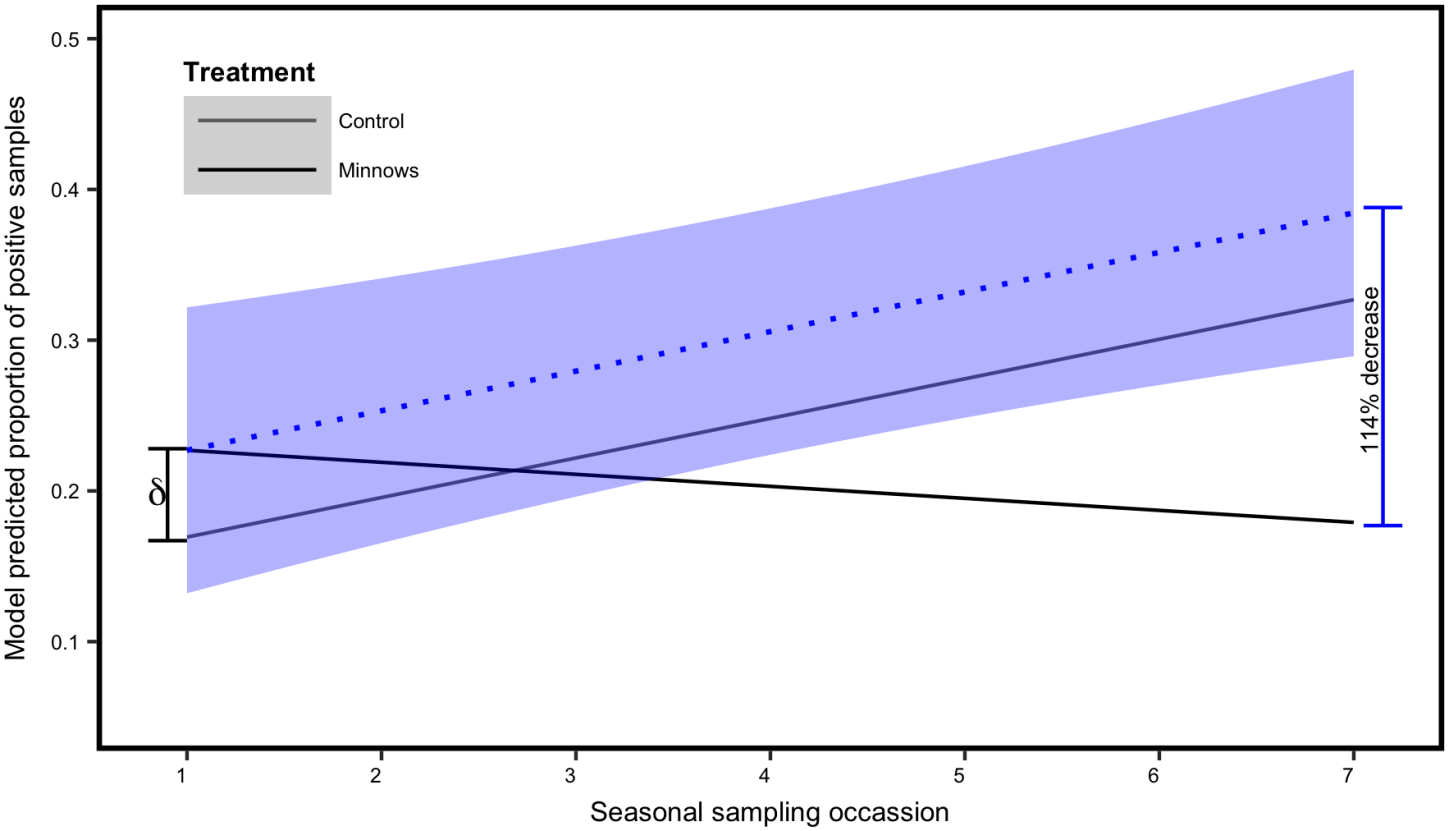
Scaled model predicted values. Predicted values from the top linear mixed effects model predicting the proportion of samples (i.e., dips) that contained either a live mosquito larva or an exoskeleton. Data were collected from 2013–2015. The black line represents the predicted values for reservoirs in which minnows were introduced (i.e., Minnows). The grey line represents reservoirs that did not contain minnows (i.e., Control). The dotted blue line represents the predicted values for the minnow-treated reservoirs scaled to have the same (higher) starting value as the Control reservoirs. The starting difference is indicated by ***δ***. This reveals a hypothetical decrease of larva abundance of 114% if minnow-treated reservoirs had started at larva densities similar to the Control reservoirs.

The top 4 mixed models accounted for the majority of explanatory power (*w*_i_ > 0.95) with the top two models accounting for the majority of the weight (*w*_i_ = 0.84; Table 2). Visual inspection of histograms of model residuals, normal Q-Q plots, and plots of residual vs. fitted values confirmed that models were well specified. The interaction of treatment and sampling occasion was included in three of the four top models (Table 2). The coefficient estimates for sampling occasion were consistently positive across the models indicating the general increase in larva abundance across the field season (Table 3). The interaction of treatment and sample had consistent coefficient estimates that did not overlap zero (Table 3). The negative coefficient estimates for the interaction term indicate decreases in the response variable with increasing sampling occasion (i.e., later in the season). Model predicted values demonstrated a consistent increase in mosquito larvae densities in control ponds throughout the season and a consistent decrease in densities within treatment ponds (Fig 4). We scaled the starting values in the control ponds (which were substantially lower than the treatment ponds due to chance) to match starting treatment pond values and predicted values from this model indicate a predicated decrease of 114% in the treatment ponds if starting larva densities had been similar (Fig 5).

**Table 2:** Top model set.

Model^a^	AIC_c_	ΔAIC_c_	Rank	*w*_*i*_
Treatment × Sample + Year	-192.0	0.00	1	0.50
Treatment + Sample + Year	-191.2	0.78	2	0.34
Treatment × Sample + Veg_cover + Year	-188.2	3.78	3	0.08
Treatment × Sample + SH + Year	-187.2	4.76	4	0.05

Top Models accounting for the top 95% of variation in the complete model set (*n* = 12) assessing the efficacy of fathead minnows for controlling mosquito population in northeast Wyoming. The response variable was the proportion of positive dips and individual ponds were included as a random intercept in all models. Variable abbreviations are defined in Table 1. All fixed effect variables (with the exception of year) were standardized prior to analysis. All models containing an interaction effect also included each individual covariate contained within the interaction. AIC_c_, Akaike’s information criterion adjusted for small sample size; ΔAIC_c_, difference in AIC from the model with the lowest AIC_c_; Rank, model rank within the set; *w*_i_, model weight within the set.

**Table 3:** Coefficient estimates.

Model	Variable	β_i_	SE
1	Treatment	0.09	0.07
	Sample	0.03	0.01
	Treatment × Sample	-0.03	0.01
	Year	-0.01	0.02
2	Treatment	-0.04	0.05
	Sample	0.01	0.01
	Year	-0.01	0.02
3	Treatment	0.04	0.06
	Sample	0.03	0.01
	Treatment × Sample	-0.03	0.01
	Veg_cover	0.04	0.02
4	Treatment	0.09	0.06
	Sample	0.03	0.01
	Treatment × Sample	-0.03	0.01
	SH	-0.04	0.02

Beta coefficients and associated standard errors for variables included in the top 95% model set. Values rounded to 2 decimal places.

Minnows survived both winters included in our 3-year study in most treatment reservoirs. During the winter of 2013–14, all reservoirs overwintered minnow populations except the two smallest that had average annual depths of 0.7m and 0.6m. During the following winter (2014–15), only one site did not have overwinter survival. Reproductive activity was observed during the summer at all treatment sites.

**Fig 6:**
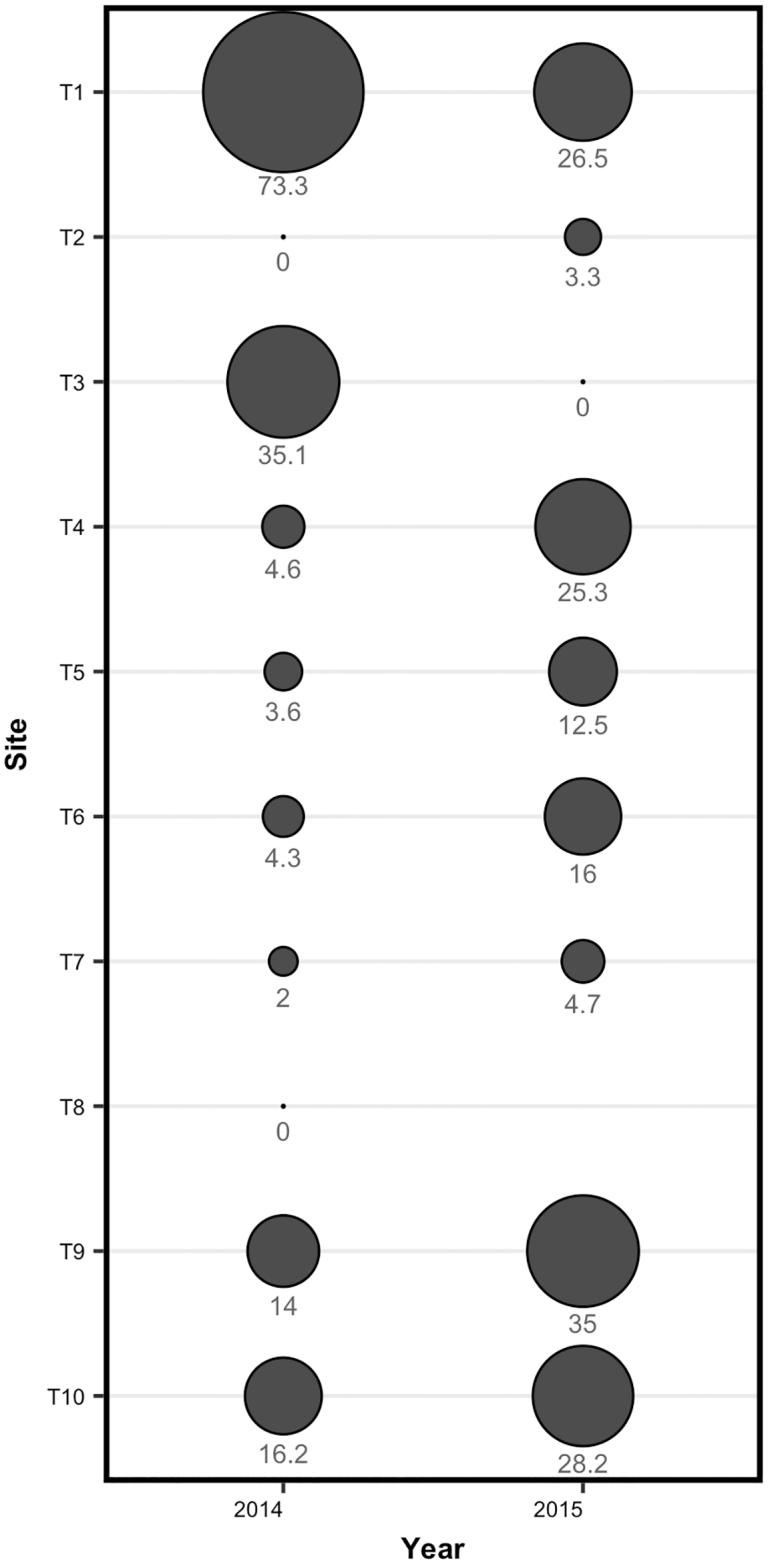
Minnow abundance. Minnow catch per unit effort (i.e., average number of minnows per transect) from electrofishing sampling in treatment ponds in 2014 and 2015. Data are presented for 10 reservoirs that contained minnows at one point during the study. Circles are sized based on the average number of minnows counted per transect.

Relative densities expressed as catch per unit effort (fish per transect) revealed increasing density between years at 7 of the 10 study sites (Fig 6). Two of the sites displayed no survival as of 2015 including T8, which was removed from the study as it did not prove to support survival in 2014. Although supporting relatively high densities in 2014, T3 dried up in 2015 and did not support fish survival. Only one site (T1) decreased in relative density while supporting survival of fathead minnows. Although T1 had identical pre-season water levels, perimeter measurements from the end of the 2015 field season revealed that it experienced significant draw-down losing 69% of its water holdings between June and August. The average summer seasonal draw-down across all sites was 36.6% (range 0–100%).

Minnow survival was not influenced by water quality. Water quality parameters (including temperature) across study sites were within tolerable ranges for fathead minnow survival (S3 Table). The top model assessing the influence of pond morphology on minnow densities accounted for 95% of the weight in the model set (*n* = 10 models) and contained estimates for depth (Beta 1.67 ± 0.57 SE) and perimeter (1.97 ± 0.54). The top model assessing the influence of water chemistry on minnow densities account for 99% of the weight in the model set (*n* = 11 models) and contained estimates for hardness (Beta 1.91 ± 0.32 SE) and sodium (-4.69 ± 0.96). As noted above, hardness was highly correlated with magnesium (*r* = 0.93) and calcium (*r* = 0.79).

## Discussion

Energy development and agricultural activities continue to increase the number of water sources within many arid landscapes, creating mesic areas that become prime breeding grounds for disease vectors such as mosquitoes. For example, between 1999 and 2004, some studies suggest a 75% increase in potential larval habitats primarily due to small coalbed methane water discharge ponds in areas such as the Powder River Basin, Wyoming [28]. Our study was the first to evaluate the efficacy of fathead minnows as a biological control agent against mosquito vectors in these environments. Importantly, we evaluated the efficacy of fathead minnows across multiple years and moisture regimes, revealing the potential to use fathead minnows as a long-term control option. We found that the mosquito species of most concern were present in our study sites. We also demonstrate that under all but the most extreme conditions, minnows establish self-sustaining populations and consume sufficient mosquito larva to cause a significant decrease in larvae densities.

Adult mosquitoes were present in both treatment and control sites. The primary vector species for WNv in Wyoming, *Culex tarsalis*, [28] was the most abundant of the 14 species identified in the samples (38%). WNv surveys from the Wyoming Department of Health reported avian, equine, and human cases of WNv in Wyoming and during all 3 years of the study period (2013–2015). In our study area, past WNv outbreaks have resulted in severe impacts to wildlife [8]. Projections of future impacts to wildlife in response to potential WNv outbreaks predict even more severe impacts [10]. WNv remains an important and largely, unaddressed, threat in semi-arid rangelands in North America.

The isotopic analyses, combined with the results from our control-treatment experiment, provided strong evidence that minnows are feeding on larvae in most cases. Although fathead minnows are opportunistic feeders, previous studies documented their capacity to suppress mosquito larva population in relatively simple ecological systems [20]. Our study system likely encompassed higher food web complexity than previous research. Although our stable isotope results were unable to determine dietary proportions, they indicated minnow predation at the mosquito larva trophic level in all but one reservoir (T7). Though we did not collect data on the abundance of other larvae in our dip samples, our many thousands of samples indicated that mosquito larvae were the most abundant prey at this trophic level in the littoral zones of the reservoirs.

The introduction of fathead minnows significantly reduced the estimated growth rate of mosquito populations through the summer. The treatment covariate was present in every model in the top 95% model set. Larvae abundance generally increased throughout the field season in control sites; however, this increase was suppressed by fathead minnows throughout the summer field season in treatment reservoirs. Larval densities were lower in controls (~50%) than in treatment reservoirs near the beginning of the field seasons purely by chance as site designation took place randomly prior to sampling. Thus, we introduced minnows into ponds with relatively high mosquito larvae abundance. Therefore, our estimates of effectiveness of fathead minnows for mosquito control are conservative. When we applied model predictions with the control sites scaled to similar starting values as the treatment sites, we estimated a 114% decrease in the proportion of positive dips in sites with minnows. We do not believe the greater larvae abundance affected minnow survival in that minnows were capable of surviving in ponds with low larvae abundance. Given the observed variation over time, it is unsurprising that the interaction between treatment and time was also present in all but one of the top models.

The outlier pond (T7) was unique in several ways. First, the near shore vegetation cover was an average of 61% across all years compared to an average of 25% at the other sites. This reservoir was deeper than all other ponds (3.42 m deeper than the combined average of 1.78 m) and had steeper banks below water level. The higher larval densities found in T7 indicated that minnows were not effective for mosquito larvae control in all situations. Minnows in this pond may be selecting for other prey in response to pond morphology conditions that either restrict access or present opportunities to feed on alternative prey sources. This hypothesis is supported by isotope data, with T7 being the only site that deviated in carbon (δ ^13^C) ratios for the fathead minnows. Previous studies have shown significant δ ^13^ C enrichment in littoral compared to pelagic consumers in lake environments [27]. We suspect the high abundance of filamentous algae and other vegetation near the shore of the pond helped protect larva from predation. We propose the existence of a threshold of pond morphology (e.g., depth) and vegetation cover affecting the efficacy of fathead minnows for mosquito control and suggest that future studies examine water bodies with a wider range of pond morphology.

Reservoir size and depth were the key features of pond morphology influencing minnow densities. During the winter of 2013–14, minnow populations survived in all but two reservoirs (T2 and T8). These reservoirs were the two smallest reservoirs (0.14 and 0.02 ha respectively) and either froze completely through in the winter, or dried up. During the following winter (2014–15), T3 was the only site in which minnows did not successfully over winter. This was likely due to significant water loss after 2014 field season, leading to minnow death as a result of complete winter freeze. Pre-field season measurements of both surface hectares and maximum depth decreased in T3 between 2014 and 2015. This reservoir also experienced rapid drawdown during the 2015 field season (100%) with no water remaining by the end of August suggesting changes in local run-off, drainage features, and water holding capacity from previous seasons. Additionally, T1 demonstrated a decrease in relative density while overwintering between years following a 69% drawdown in water during the 2015 field season. Overwinter dissolved oxygen (DO) levels in reservoirs with winter sampling access proved to be suitable for fathead minnow survival with an overall average of 6.35 mg·L^-1^ [30,31].

We measured multiple water quality parameters (*n* = 13) that could affect minnow population densities through influences on reproduction or survival [29]. Hardness and sodium had the greatest influence on minnow population densities. However, it is important to note that all water quality measures were within tolerable ranges and overall survival was high. Hardness had a positive estimated coefficient and sodium (highly correlated with magnesium and calcium) had a negative estimated coefficient suggesting that increases in sodium would lead to lower minnow densities. Reservoir conditions in northeast Wyoming can change annually and could extend beyond acceptable ranges for fathead minnows. Therefore, it may be prudent to assess water quality prior to the introduction of fathead minnows in reservoirs.

## Conclusions and recommendations

We caution that fathead minnows are not a panacea for mosquito larvae control in North American semi-arid rangelands. As with all biological control efforts, there are risks associated with the introduction of novel predators into a system. In particular, stocking fathead minnows in interconnected water systems must be done with extreme caution. In high abundance, the fathead minnow has the potential to significantly influence aquatic ecosystems [22]. For example, small bodied fishes such as the fathead minnow have the potential to suppress Wood frog (*Rana sylvatica*) populations in Boreal Alberta lakes [32]. Other research has found that fathead minnows can have an influence on multiple biotic factors in prairie pothole wetlands, including abundance of aquatic insects and salamanders [33,34]. The sites used in our study were all eutrophic water bodies used as cattle reservoirs where turbidity and nutrient levels had extremely high baselines and were isolated from streams and other water bodies.

Fathead minnows proved to be an effective method of control for mosquito larvae population densities. Fathead minnows also demonstrated the ability to suppress temporal variation in larvae abundance. Northeast Wyoming has a large number of livestock reservoirs, drainages, and other man-made water sources which represent the largest potential breeding sites for mosquito populations in this landscape. Controlling mosquito larval populations is crucial for reducing the impacts of WNv, since the mesic areas created by reservoirs provide habitat that attracts and concentrates populations of sage-grouse. As WNv could potentially be one of the primary factors impacting sage-grouse populations in northeastern Wyoming, these environments become even more important for mosquito control [35]. Although some water sources may not be suitable for fathead minnow introduction, many of the reservoirs in rural northeastern Wyoming are comparable to those featured in this study and would be prime targets for minnow introduction as part of larger control efforts.

To facilitate successful implementation, treatment site selection must be considered carefully. Reservoirs should be surveyed prior to minnow introduction to determine species presence, morphology, vegetation coverage, and water quality. Two critical research gaps remain: 1) how effective is this treatment across a broader range of pond morphology and site conditions (e.g., depth, benthic gradients, water quality) and 2) what are the potential landscape-level impacts of broad-scale implementation of this strategy? Integration with other potential management options may create optimal outcomes for widespread control of mosquito populations and WNv. Regardless of project size, economic costs must also be considered alongside fathead minnow efficacy. The low financial cost of stocking fathead minnows in livestock reservoirs compared to other forms of mosquito control (e.g., larvicide pucks, aerial spraying) should facilitate the implementation of this control strategy.

We recommend carefully considered and strategic introduction of minnows in isolated reservoirs in high priority brood rearing areas. These sites could be identified using spatial predictions of brood rearing habitat as those identified in [36].

## Supporting information

S1 Table Reservoir characteristics. Reservoir size, morphology and vegetation measures for all 3 study seasons from 2013–2015 including stocking rates.
(PDF)

S2 Table Winter dissolved oxygen. Winter 2014 Dissolved oxygen (mg·L^-1^) levels between January 24, 2014 and February 10, 2014. Measurements were only taken at sites with available road access. Ice sheet thickness was between 0.5 and 0.6 meters for all sites. This thickness was included in depth marks for DO readings.
(PDF)

S3 Table Water quality. Water quality indicators measured in the field and laboratory. Values include the mean and standard deviation for treatment sites included in this study, with optimal range and maximum or minimum tolerances obtained from the literature on fathead minnows or closely related species. A value of ND indicates no data exist for a particular parameter.
(PDF)
