# Supplementary material for: Assessing the efficacy of fathead minnows (Pimephales promelas) for mosquito control
Source: PLoS One. 2018 Apr 12;13(4):e0194304. doi: 10.1371/journal.pone.0194304 (PMC5896899; doi:10.1371/journal.pone.0194304)
Supplement: S1 Table — Reservoir size, morphology and vegetation measures for all 3 study seasons from 2013–2015 including stocking rates. (PDF) [file pone.0194304.s001.pdf]

S1 Table. Reservoir Characteristics. Reservoir size, morphology and vegetation measures for all 3 study seasons from 2013-2015 including stocking rates.

	Max Depth (m)			Emergent Vegetation (% cover)			Size (ha)			Minnows Stocked	
**Pond ID**	**2013**	**2014**	**2015**	**2013**	**2014**	**2015**	**2013**	**2014**	**2015**	**2013**	**2014**
**C1**	2.8	2.8	2.8	12.9	20.7	46.3	0.92	0.92	0.92	0	0
**C2**	0.6	0.6	0.6	0	0	0	0.22	0.22	0.22	0	0
**C3**	0.3	1.5	1.5	0	0	0	0.13	0.40	0.40	0	0
**C4**	2.2	2.2	2.5	0	6.7	0	0.07	0.07	0.16	0	0
**C5**	1.3	0.9	1.3	0	61.8	2.9	0.42	0.10	0.77	0	0
**C6**	0.8	0.8	1.1	11.3	22.7	75.8	0.47	0.47	0.67	0	0
**T1**	2.1	2.1	2.1	0	62.5	32.0	1.33	1.33	1.33	8,200	8,200
**T2**	0.4	1.3	0.4	0	36.3	34.5	0.14	0.43	0.43	1,000	1,500
**T3**	1.8	1.4	1.1	0	20.3	2.9	0.63	1.16	0.63	3,900	6,400
**T4**	1.1	1.1	1.1	0	0	0	0.38	0.38	0.38	2,400	2,400
**T5**	1.2	1.2	1.2	0	34.0	63.0	0.91	0.91	0.91	5,700	7,500
**T6**	1.5	1.5	2.1	48.6	14.6	9.1	0.31	0.31	0.40	2,000	2,000
**T7**	5.2	5.2	5.2	46.8	36.9	100	0.22	0.22	0.22	1,500	1,500
**T8**	0.6	0.6	na	100	100	na	0.02	0.02	na	500	500
**T9**	na	1.5	1.5	na	0	33.3	0.26	0.26	0.26	0	2,500
**T10**	2.3	2.4	2.4	58.3	47.3	57.6	0.55	0.55	0.46	0	0
									**Total**	**25,200**	**32,500**
