# Supplementary material for: Assessing the efficacy of fathead minnows (Pimephales promelas) for mosquito control
Source: PLoS One. 2018 Apr 12;13(4):e0194304. doi: 10.1371/journal.pone.0194304 (PMC5896899; doi:10.1371/journal.pone.0194304)
Supplement: S2 Table — Winter 2014 Dissolved oxygen (mg·L-1) levels between January 24, 2014 and February 10, 2014. Measurements were only taken at sites with available road access. Ice sheet thickness was between 0.5 and 0.6 meters for all sites. This thickness was included in depth marks for DO readings. (PDF) [file pone.0194304.s002.pdf]

S2 Table. Winter Dissolved Oxygen. Winter 2014 Dissolved oxygen ($mg \cdot L^{-1}$) levels between January 24, 2014 and February 10, 2014. Measurements were only taken at sites with available road access. Ice sheet thickness was between 0.5 and 0.6 meters for all sites. This thickness was included in depth marks for DO readings.

Site	Surface	0.3m	0.6m	0.9m	1.2m	1.5m	1.8m	2.1m	2.4m
Baseline	10.5	4.1	3.4	2.6	2.3	2.1	2	2	na
T1	11.1	10.9	10.9	6.4	2.4	2	na	na	na
T10	9.2	8.9	8.8	8.3	5	4.5	3.8	na	na
T6	5.6	5.7	6.8	2.6	na	na	na	na	na
T7	11.2	11.3	11.3	10.4	10.1	9.8	8.1	7.3	6.2
Average	9.5	8.2	8.2	6.1	5.0	4.6	4.6	4.7	6.2
