# Supplementary material for: Assessing the efficacy of fathead minnows (Pimephales promelas) for mosquito control
Source: PLoS One. 2018 Apr 12;13(4):e0194304. doi: 10.1371/journal.pone.0194304 (PMC5896899; doi:10.1371/journal.pone.0194304)
Supplement: S3 Table — Water quality indicators measured in the field and laboratory. Values include the mean and standard deviation for treatment sites included in this study, with optimal range and maximum or minimum tolerances obtained from the literature on fathead minnows or closely related species. A value of ND indicates no data exist for a particular parameter. (PDF) [file pone.0194304.s003.pdf]

S3 Table. Water Quality. Water quality indicators measured in the field and laboratory. Values include the mean and standard deviation for treatment sites included in this study, with optimal range and maximum or minimum tolerances obtained from the literature on fathead minnows or closely related species. A value of NA indicates no data exist for a particular parameter.

	Reservoir		Tolerances		
**Parameter**	**Mean ± SD**	**Optimal Range**	**Min**	**Max**	**References**
Water Temperature (°C)	22.8 ± 3.6	20.9 – 29°C	NA	40.2	[1,2]
Dissolved Oxygen (mg·L^{-1})	8.5 ± 3.5	>7 mg·L^{-1}	1-4	NA	[1,3,4]
pH	8.7 ± 1.4	7.4 - 8.2	6	NA	[5,6]
Conductivity (µs)	1284 ± 985	NA	NA	NA	
Total Alkalinity (as CaCO3) (mg·L^{-1})	104.2 ± 38.2	42 mg·L^{-1}	NA	2000	[1]
Hardness	413.7 ± 239.5	40 - 300 mg·L^{-1}	NA	NA	[1]
Ammonia Nitrogen	0.08 ± 0.20	< 2.19 mg·L^{-1}	NA	2.19[a]	[7]
Calcium (mg·L^{-1})	76 ± 40.8	NA	NA	300-1000	[8]
Magnesium (mg·L^{-1})	54.6 ± 41.5	NA	NA	100-400	[8]
Sodium (Na^+, mg·L^{-1})	80.8 ± 138.6	NA	NA	8000	[9]
Dissolved Iron (mg·L^{-1})	0.07 ± 0.17	NA	NA	1	[8]
Dissolved Manganese (mg·L^{-1})	0.01 ± 0.03	NA	NA	50	[8]
Total Phosphorus (mg·L^{-1})	0.17 ± 0.39	NA	NA	NA	[8]

[a]Maximum tolerance of Ammonia Nitrogen before experiencing negative effects on reproduction.

1. Denny JS (1987) Guidelines for the culture of fathead minnows *Pimephales promelas* for use in toxicity tests. Duluth, Minnesota.

2. Lyons J, Zorn T, Stewart J, Seelbach P, Wehrly K, et al. (2009) Defining and characterizing coolwater streams and their fish assemblages in Michigan and Wisconsin, USA. J Fish Manag 29: 1130–1151.

3. Brungs W (1971) Chronic effects of constant elevated temperature on the fathead minnow (Pimephales promelas rafinesque). Trans Am Fish Soc 100: 659–664.

4. Igram R, Wares WD (1979) Oxygen consumption in the fathead minnow (*Pimephales promelas rafinesque*)—II effects of pH, osmotic pressure, and light level. Comp Biochem Physiol Part A Physiol 62: 895–897. doi:10.1016/0300-9629(79)90024-0.

5. Mount D (1973) Chronic effect of low pH on fathead minnow survival, growth and reproduction. Water Res 7: 987–993.

6. Rahel FJ, Magnuson JJ (1983) Low pH and the absence of fish species in naturally acidic Wisconsin lakes: inference for cultural acidification. Can J Fish Aquat Sci 40: 3–9.

7. Armstrong BM, Lazorchak JM, Murphy C a, Haring HJ, Jensen KM, et al. (2012) Determining the effects of ammonia on fathead minnow (*Pimephales promelas*) reproduction. Sci Total Environ 420: 127–133. doi:10.1016/j.scitotenv.2012.01.005.

8. Doudoroff P, Katz M (1953) Toxicity of industrial wastes and their components to fish. Sew Ind Waste 25: 802–839.

9. Burnham BL, Peterka JJ (1975) Effects of saline water from North Dakota lakes on survival of fathead minnow (*Pimephales promelas*) embryos and sac fry. J Fish Res Board Canada 32: 809–812. doi:10.1139/f75-107.
